# The Role of Atypical Protein Kinase C in CSF-1-Dependent Erk Activation and Proliferation in Myeloid Progenitors and Macrophages

**DOI:** 10.1371/journal.pone.0025580

**Published:** 2011-10-18

**Authors:** Angel W. Lee

**Affiliations:** 1 Department of Pharmacology, The University of Michigan Medical School, Ann Arbor, Michigan, United States of America; 2 Center for Proteomics and Systems Biology, The Institute of Molecular Medicine at The University of Texas Health Sciences Center, Houston, Texas, United States of America; 3 OncProTech, Ann Arbor, Michigan, United States of America; Ohio State University, United States of America

## Abstract

Colony stimulating factor-1 (CSF-1 or M-CSF) is the major physiological regulator of the proliferation, differentiation and survival of cells of the mononuclear phagocyte lineage. CSF-1 binds to a receptor tyrosine kinase, the CSF-1 receptor (CSF-1R). Multiple pathways are activated downstream of the CSF-1R; however, it is not clear which pathways regulate proliferation and survival. Here, we investigated the role of atypical protein kinase Cs (PKCζ) in a myeloid progenitor cell line that expressed CSF-1R (32D.R) and in primary murine bone marrow derived macrophages (BMMs). In 32D.R cells, CSF-1 induced the phosphorylation of PKCζ and increased its kinase activity. PKC inhibitors and transfections with mutant PKCs showed that optimal CSF-1-dependent Erk activation and proliferation depended on the activity of PKCζ. We previously reported that CSF-1 activated the Erk pathway through an A-Raf-dependent and an A-Raf independent pathway (Lee and States, *Mol. Cell. Biol.* **18**, 6779). PKC inhibitors did not affect CSF-1 induced Ras and A-Raf activity but markedly reduced MEK and Erk activity, implying that PKCζ regulated the CSF-1-Erk pathway at the level of MEK. PKCζ has been implicated in activating the NF-κB pathway. However, CSF-1 promoted proliferation in an NF-κB independent manner. We established stable 32D.R cell lines that overexpressed PKCζ. Overexpression of PKCζ increased the intensity and duration of CSF-1 induced Erk activity and rendered cells more responsive to CSF-1 mediated proliferation. In contrast to 32D.R cells, PKCζ inhibition in BMMs had only a modest effect on proliferation. Moreover, PKCζ -specific and pan-PKC inhibitors induced a paradoxical increase in MEK-Erk phosphorylation suggesting that PKCs targeted a common negative regulatory step upstream of MEK. Our results demonstrated that CSF-1 dependent Erk activation and proliferation are regulated differentially in progenitors and differentiated cells.

## Introduction

Colony stimulating factor-1 (CSF-1 or M-CSF) is a growth factor secreted by numerous cell types, whose synthesis is often increased in response to different stimuli such as those causing inflammation [1]. It promotes the proliferation, survival and differentiation of cells of the mononuclear phagocyte (MNP) lineage and their myeloid progenitors [1], [2]. CSF-1 acts on the CSF-1R, a receptor tyrosine kinase (RTK) of the platelet-derived growth factor (PDGF) receptor family that also includes c-*kit* and the Flt3/Flk2 receptor. CSF-1R, c-Kit and Flt3 all play pivotal roles in hematopoiesis. The importance of CSF-1-CSF-1R signaling *in vivo* is revealed by the pleiotropic functional defects of the CSF-1 null (*op/op*) and CSF-1R null mice [1], [2] including deficiencies in hematopoietic progenitors, monocytes, macrophages, innate immune responses, bone remodeling, mammary gland development and fertility. CSF-1-CSF-1R signaling contributes to the inflammatory component associated with many chronic diseases, notably cancer, arthritis, neurodegenerative diseases, atherosclerosis and obesity. Oncogenic signals emanating from an aberrantly expressed CSF-1R in tumor cells promote tumor growth and metastasis in breast and ovarian cancers [3]. A second ligand for the CSF-1R was recently identified [4], IL-34, that has a different spatiotemporal expression pattern from CSF-1 [5] and is likely to regulate microglial development in the brain [6]. The existence of IL-34 explains the milder phenotype of CSF-1 null mice compared to mice lacking CSF-1R.

CSF-1 binding initiates dimerization of the CSF-1R and results in kinase activation [7], [8], autophosphorylation, recruitment of signaling molecules to the receptor, and rapid stimulation of downstream signaling. Tyrosine autophosphorylation sites mapped in the CSF-1R are Tyr 697, Tyr 706 and Tyr 721 in the kinase insert region that divides the catalytic domain, Tyr 807 in the activation loop of the catalytic domain, Tyr 559 in the juxtamembrane region, and Tyr 921, Tyr 974 in the carboxy-terminus [1], [9]. Many molecules have been reported to bind these autophosphorylation sites. Of note, Tyr 559 has a dual function: when not phosphorylated, it has an autoinhibitory role, when phosphorylated, it binds Src family kinases (SFK) [10], [11]. Tyr 697 and Tyr 921 bind Grb2 [12], [13], an adaptor that is constitutively associated with the Ras guanine nucleotide exchange factor (Sos). Membrane localization of Ras leads to activation of the Raf-MEK-Erk (extracellular signal regulated kinase)/MAPK (mitogen activated protein kinase) pathway [14]. Tyr 721 binds the p85 regulatory subunit of phosphatidylinositol 3-kinase (PI3K) [15] bringing the lipid kinase into proximity to its substrates in the plasma membrane. Additionally, the CSF-1R recruits PI3K via an alternate pathway involving SFKs and Gab2 [16]. Tyr 974 binds c-Cbl [17], an E3 ubiquitin ligase important for CSF-1R downregulation and termination of receptor signaling [18]. CSF-1 binding also stimulates the tyrosine phosphorylation of other molecules such as SHC and Ship [1] and together the receptor signaling complex mediates MNP development and mature cell function and activation.

The Ras-Raf-MEK-Erk pathway is evolutionarily conserved and controls many aspects of cell activities [14]. We previously showed that CSF-1 induced Erk activity is important for supporting cell survival in a myeloid progenitor cell line, 32D.R [19]. In these cells, A-Raf and not c-Raf-1 mediates a part of the activating signal from the CSF-1R to the Erk kinase, MEK1; PI3K is also upstream of MEK1 but working in parallel to A-Raf [16]. How PI3K signals to MEK1 is not clear since it is a lipid kinase and MEK1 requires phosphorylation at two serines for activation. Potentially, PI3K could target an inhibitor of the pathway. For example, Raf kinase inhibitor protein (RKIP) binds to c-Raf-1 and prevents MEK phosphorylation [14] but does not appear to affect B-Raf activation [20] and its influence on A-Raf is unknown. Another possibility is input from the protein kinase C (PKC) family [21]. Members of the PKC family belong to one of three classes: conventional (PKCα, βI, βII, γ), novel (δ, ε, θ, η) and atypical (ζ, λ) [22]. PKCs are regulated by a series of priming phosphorylations [22], including phosphorylation in the active site by 3′-phosphoinositide-dependent kinase-1 (PDK-1), in the turn motif by the mTORC2 complex and in conventional and novel PKCs in the hydrophobic motif by different kinases, including mTORC2. These phosphorylations play important roles in stabilizing PKCs. Conventional PKCs bind to calcium and diacylglycerol and are recruited to the plasma membrane whereas novel PKCs bind only to diacylglyerol; atypical PKCs do not bind either calcium or diacyglycerol. The interactions with the plasma membrane result in a conformational change that leads to downstream signaling. All three PKC classes can stimulate the Erk pathway but only conventional and novel PKCs activate MEK-Erk via c-Raf-1 whereas atypical PKCs such as PKCζ use a c-Raf-1 independent mechanism [23]. PKCζ -dependent Erk activity is necessary for NF-κB activation [24]. Depending on cell type however, PKCζ deficiency does not always result in diminished Erk activation [25]. PI3K is reported to increase PKCζ activity by enhancing Thr 410 phosphorylation in a PDK-1-dependent manner [26]. Previous work examining the role of PKCζ in CSF-1 signaling had yielded controversial findings. In one study, CSF-1 was reported to increase the *in vitro* autokinase activity of a catalytic fragment of PKCδ but activated PKCζ was not detected in that assay [27]. In another study, PKCζ activation by CSF-1 was assessed by membrane translocation [28], but that may not be an adequate indication of PKCζ activation since atypical PKCs are not dependent on diacylglcyerol generated at the membrane for activation. Yet in a third study PKCζ knockdown was found to reduce CSF-1 induced macrophage migration [29].

Herein we tested the hypothesis that PKCζ may mediate the A-Raf independent pathway to activate MEK-Erk in response to CSF-1 in myeloid cells: 32D.R myeloid progenitors and primary bone marrow derived macrophages (BMMs). We found that CSF-1 increased PKCζ Thr 410 phosphorylation and kinase activity in 32D.R cells. Pharmacologic inhibition and transfection studies demonstrated that atypical PKCs but not conventional or novel PKCs contributed towards CSF-1 induced MEK-Erk activity in a c-Raf-1 and A-Raf-independent fashion. While PKCζ kinase inhibition reduced CSF-1 supported mitogenesis in 32D.R cells, overexpression of PKCζ increased CSF-1 mitogenic responsiveness. However, PKCζ's promotion of mitogenic signaling in 32D.R cells was independent of NF-κB. In BMMs, PKCζ inhibition had a more modest effect on CSF-1 dependent mitogenesis, and, pan-PKC inhibition had a paradoxically enhancing effect on MEK-Erk phosphorylation. Thus the importance of PKCζ in the control of CSF-1 mediated MEK-Erk activity and mitogenesis depends on differentiation stage.

## Methods

### Antibodies and reagents

Cell culture reagents and media were from Life Technologies (Carlsbad, CA) or Sigma-Aldrich (St. Louis, MO). GF109203X was from EMD Chemicals (Rockland, MA) or Enzo Life Sciences (Plymouth Meting, PA), Ro-31-8220 was from Axxora (San Diego, CA) and Go 6983 was from EMD Chemicals. Myelin basic protein (MBP) was from Life Technologies, PKCε pseudosubstrate peptide (residues 149–164, Ala to Ser 159) as phosphorylation substrate and myristoylated PKCζ pseudosubstrate peptide were from Enzo Life Sciences. Recombinant human CSF-1 was a gift of Genetics Institute (Cambridge, MA), recombinant murine interleukin-3 (IL-3) was from Life Technologies, and phorbol 12-myristate 13-actetate (PMA) was from EMD Chemicals.

Polyclonal antibodies against c-Raf-1, A-Raf, Erk2, were from Santa Cruz Biotechnology (Santa Cruz, CA). Antibodies against PKCα, PKCβ, PKCγ, PKCδ and PKCε were from Life Technologies. We used a rabbit polyclonal antibody against PKCζ for immunoprecipitations or a monoclonal antibody for immunoblotting (both from Santa Cruz). The following monoclonal antibodies were used: MEK1 from BD Transduction Labs (Lexington, KY), Myc (9E10) from Santa Cruz, hemagglutinin (HA) antibody from BAbCo (Berkeley, CA), and Ras Ab-4 from EMD Chemicals. Phosphospecific antibodies that recognize Erk or MEK were from Cell Signaling Technology (Danvers, MA) and an antibody that recognizes Thr 410 of PKCζ was a gift from Alex Toker (Harvard Medical School) or purchased from Santa Cruz.

### Animals

A colony of C57BL/6 mice was housed in a specific pathogen-free environment. The Animal Welfare Committee at the University of Texas Health Science Center, Houston approved all animal protocols (IACUC assurance number: A3413-01, protocol number 08-131 and 09-032) and studies were carried out in accordance with the recommendations in the Guide for the Care and Use of Laboratory Animals of the National Institutes of Health. Mice were sacrificed by CO_2_ asphyxiation followed by cervical dislocation.

### Plasmids

PKC constructs utilized in this study were as follows: PKCζ (T/A)_4_, obtained from Peter Parker (ICRF, London), is a dominant-negative PKCζ with Thr→Ala substitutions at the activation loop phosphorylation sites [30]; constitutively active HA-tagged PKCζ, consisting of only the catalytic domain of PKCζ [31] was from Jorge Moscat (Universidad Autonoma de Madrid, Madrid). PKC constructs used in transient transfections were cloned into the expression vector pcDNA3 (Invitrogen). For stable transfections, wildtype PKCζ was cloned into pEFIRES-puro [32]. The construction of Myc-tagged Erk2 has been described [16]. The NF-κB reporter plasmid (pBxVIII) containing 6 tandem κB binding sites was obtained from Gabriel Nunez (University of Michigan Medical School) [33].

### Recombinant proteins

Recombinant bacterially-produced His-tagged kinase-dead MAPK, MEK and GST-RBD, containing the Ras binding domain of Raf (residues 51–131) fused to GST (plasmid generously provided by Johannes Bos, Utrecht University, Utretcht) were expressed and purified as described [16].

### Cell culture and transfections

The IL-3-dependent myeloid progenitor cell line expressing CSF-1R (32D.R) has been described previously [19]. A stable 32D.R cell line expressing an NF-kB luciferase reporter was established by co-transfection with pBxVIII and pPUR (Clontech Laboratories, Mountain View, CA) and mass populations selected in 1 µg/mL puromycin. A stable 32D.R cell line expressing WT- PKCζ was similarly established by electroporation with pEFIRES-p-WT- PKCζ. BMMs were produced as described previously [34]. Briefly, total bone marrow cells were isolated from the femurs and tibias of C57BL/6 mice and induced to differentiate to macrophages over 7 days. For stimulation experiments, exponentially growing 32D.R cells were washed thoroughly in Hank's Buffered Salt Solution and deprived of serum and IL-3 for 3 h prior to treatment with inhibitors and growth factors. For BMMs, on day 7, cells were thoroughly washed and starved in the absence of CSF-1 for 12 hours. Cells were then pretreated with inhibitors for 1 h followed by CSF-1 addition. The protocol for transient transfection has been previously described [19] and transfected cells were allowed to express recombinant proteins for 24 h before starvation and analysis.

### Immunoprecipitation, immunoblotting, kinase assays and Ras activation assay

Protein analysis methods have been extensively described in previous publications [16], [19], [34]. To measure the kinase activity of PKCζ, PKCζ was immunoprecipitated from cell lysates and subjected to an *in vitro* kinase assay in the presence of 10 µCi of γ^32^P ATP, 50 µM ATP and 50 µM PKCε peptide as substrate. Reaction proceeded for 8 min at 30°C and was spotted onto P81 phosphocellulose paper as described previously [19] 1 µM Ro-31-8220 was included in the kinase reaction in parallel samples to confirm the phosphorylation was due to PKCζ. ^32^P incorporation was quantified using a Storm PhosphorImager (Molecular Dynamics). Immunoblots were detected using ECL or ECL plus (GE Healthcare). To quantify band intensities on immunoblots, multiple exposures of each blot were obtained and band intensities determined using the NIH Image 1.43 software except where noted in the figure legend.

### Subcellular fractionation

Cells were resuspended at a density of 8×10^7^/ml in HB (10 mM β-glycerophosphate pH 7.5, 10 mM KCl, 1 mM EDTA, 1 mM EGTA, 1 mM MgCl_2_) supplemented with protease and phosphatase inhibitors and homogenized with a Dounce homogenizer until cell breakage (monitored by phase-contrast microscopy and Trypan Blue staining) was >90%. Nuclei and unbroken cells were removed by centrifugation at 1000×g for 5 min and the supernatant re-centrifuged at 100,000×g for 1 hr. The supernatant was adjusted to contain a final concentration of 0.5% NP40, 0.1% sodium deoxycholate and 0.1% Brij35 and after spinning, designated the S100 (cytosolic) fraction. The pellet after the 100,000×g spin was Dounce homogenized and extracted with HB containing the above detergents, centrifuged at 16,000×g for 15 min to remove insoluble material and the supernatant designated the P100 fraction. Variation in the ratio of total cytosolic to membrane protein amongst the samples was 3% indicating equivalent fractionation and extraction. Purity of the fractions was determined by assaying for the activity of a cytosolic marker, lactate dehydrogenase, and for the presence of Ras, by Western blotting. Less than 10% of the LDH activity was found in the membrane (P100) fraction and no Ras was detected in the cytosolic (S100) fraction (data not shown).

### Proliferation assay

Proliferation was assessed using the MTS tetrazolium assay (CellTiter 96™kit, Promega, Madison, WI) as described [19]. 3,000 32D.R cells or 3,750 BMMs were plated in triplicate in 96 well plates in the absence or presence of inhibitors or growth factors. MTS activity was read at 48 h unless otherwise stated. Cell counts were performed in duplicate using a hemocytometer and dead cells excluded with Trypan Blue.

### Luciferase assay

Luciferase activity was assayed using the Luciferase Reporter Assay (Promega). 32D.R cells stably expressing an NF-κB reporter were washed extensively and starved in serum-free medium [19] for 2 h before the addition of inhibitors, followed 1 h later by 5 nM CSF-1 or 20 ng/mL TNFα. Cells were harvested 6 h later. For long-term activation, cells were starved in media without growth factors for 6 h, before the addition of inhibitors, followed by CSF-1 or TNFα. 10 µg (CSF-1 series) or 5 µg (TNFα) of lysate was used and each assay performed in duplicate.

## Results

### Inhibition of atypical PKC activity decreases CSF-1 dependent mitogenesis in 32D.R myeloid progenitors

**Figure 1:**
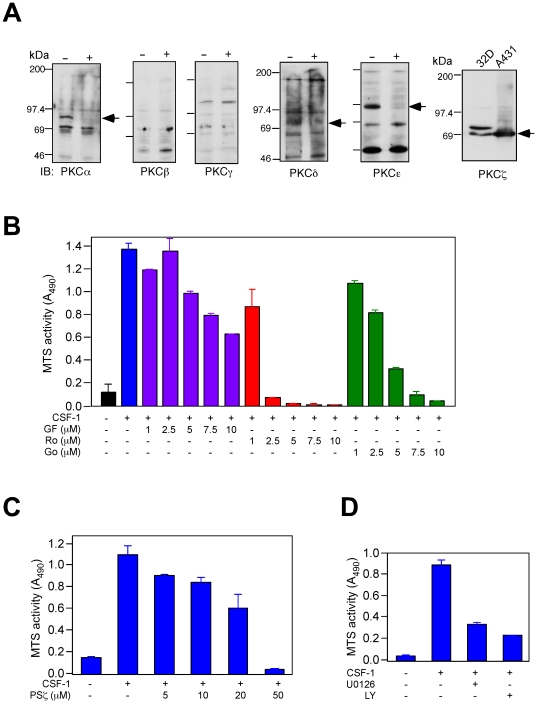
The effect of PKC inhibitors on CSF-1 dependent mitogenesis. (**A**) PKC immunoblots. “+” refers to the presence of a competing peptide. Arrow points to the PKC band that is competed off by the corresponding peptide. The lower band in the PKCζ blot is the authentic PKCζ protein (see text). (**B**) MTS assay. 32D.R cells were plated under the conditions shown and in the presence of 5 nM CSF-1. MTS activity was assayed after 48 h. (**C**) MTS assay was performed as described in (A) in the presence of the indicated amounts of PKCζ pseudosubstrate peptide. (**D**) MTS assay in the presence of CSF-1 and the indicated inhibitors, 10 µM U0126 and 5 µM LY294002.

32D.R cells express cell surface markers (CD31^+^Ly6C^+^CD11b^−^) that are consistent with an immature myeloid phenotype [35] (**Fig. S1**, and Methods S1). We have previously shown that Erk activity is important for CSF-1 supported proliferation and survival in these cells [19]. To determine if PKC has a similar role in promoting CSF-1 mitogenesis, we first determined the expression pattern of PKCs. 32D.R cells expressed PKCα, PKCδ, PKCε and PKCζ but not PKCβ or PKCγ (**Fig. 1A**). Two bands around 70 kDa were detected in the PKCζ blot. We believe the lower band to be the authentic PKCζ protein because only the lower band was present in A431 cells (positive control provided by the manufacturer), it was not affected by chronic phorbol ester treatment (see later) nor induced to translocate to the membrane (see later). Lastly, it co-migrated with transiently overexpressed PKCζ (see later).

To assess the role of PKCs in CSF-1 dependent growth we used the MTS assay [19], [34]. We tested the effect of PKC inhibitors with different specificities: GF109203X (also known as Bisindolylmaleimide I, or Gö 6850) inhibits purified conventional and novel PKCs in the nanomolar range and atypical PKCs in the micromolar range [36]. Gö 6893 inhibits purified conventional and novel PKCs at <10 nM and PKCζ at 60 nM [37]. Ro 31-8220 is a potent inhibitor of PKCs but has been reported to inhibit non-PKC kinases using *in vitro* assays [38] although this observation may not be applicable to intact cells [39]. The PKCζ pseudosubstrate peptide mimics the substrate binding site in PKCζ but with a non-phosphorylatable Ala in place of the target site and is specific for PKCζ [40]. In 32D.R cells, GF109203X inhibited CSF-1 dependent mitogenesis with an IC_50_ of ≈7.5 µM whereas the IC_50_ for Gö 6983 was between 2.5 and 5 µM (**Fig. 1B**). At 2.5 µM, Ro-31-8220 completely prevented CSF-1 dependent mitogenesis. At 1 µM, both GF109203X and Gö 6983 should have inhibited conventional and novel PKCs, but the reduction in CSF-1 dependent mitogenesis was minimal. The IC_50_ of ≈7.5 µM for GF109203X is consistent with a role for PKCζ yet at the concentration that is reported to inhibit PKCζ (1 µM), Gö 6983 was less potent. However published IC_50_ values are based on inhibition of recombinant PKC isoenzymes and do not reflect accessibility to intracellular stores of these enzymes, or duration of inhibition which in turn would depend on factors such as reversibility of inhibition, half life of the inhibitor and protein turnover. We also used a myristoylated PKCζ pseudosubstrate peptide to directly investigate the role of PKCζ. A dose dependent effect was observed and the IC_50_ was ≈20 µM, which is in the range of what has been reported in published work [29], [41]. For comparison, MEK and PI3K inhibition by U0126 and LY294002 respectively markedly reduced CSF-1 supported mitogenesis (**Fig. 1C, D**). Collectively these results suggest the possibility that PKCζ contributes to CSF-1 dependent growth in 32D.R cells.

### PKC inhibitors reduce CSF-1-stimulated MEK-Erk activity in a pathway parallel to Raf and downstream of Ras

**Figure 2:**
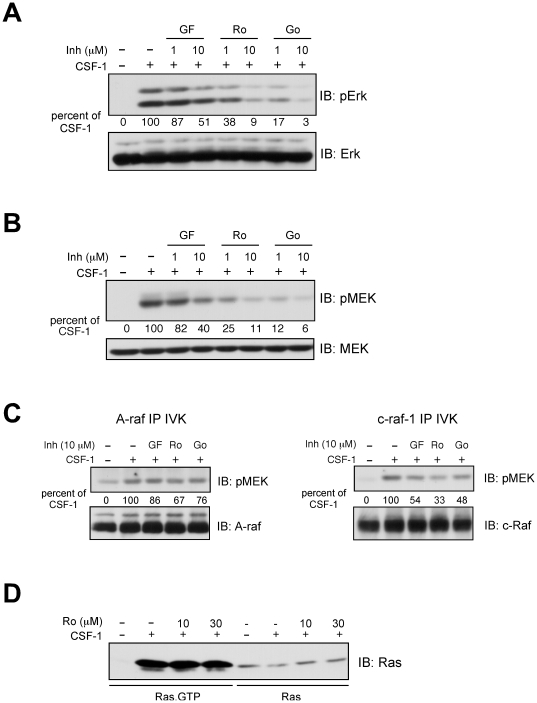
The effect of PKC inhibitors on CSF-1 mediated Erk, MEK, Raf and Ras activation. (**A**, **B**) 32D.R cells were pretreated with the indicated amounts of inhibitors (Inh) followed by CSF-1 stimulation for 4 min. Lysates were immunoblotted with phospho-Erk (top) or total Erk1/2 (bottom) antibody (**A**) or with phospho-MEK (top) and total MEK1 (bottom) antibody (**B**). (**C**) Cells were processed as described for (A, B). Inhibitors were added at 10 µM. Lysates were immunoprecipitated with either A-Raf or Raf-1 antibody and immune complexes used in an *in vitro* kinase (IVK) assay with KD-MEK as substrate. Phospho-MEK was then detected by immunoblotting. In (A–C), changes in phosphorylation are expressed as percentages of CSF-1 mediated increase in phosphorylation over unstimulated cells, where 0% and 100% denote phosphorylation in the absence or presence of CSF-1 without inhibitors. (**D**) Ras activation. Cells were starved and pretreated as indicated with Ro-31-8220 (10 µM or 30 µM) before stimulation with 5 nM CSF-1 for 4 min. Ras-GTP was extracted from 500 µg of lysates using the GST-RBD pull-down assay. 50 µg of total cell lysates were also loaded for comparison.

We have previously shown that CSF-1 stimulates the MEK-Erk pathway by both an A-Raf dependent and A-Raf independent pathway [16]. We hypothesized that PKC may mediate the A-Raf independent pathway. Pretreatment with GF109203X, Ro-31-8220 or Gö 6983 all reduced CSF-1 induced Erk (**Fig. 2A**) and MEK (**Fig. 2B**) phosphorylation. Since GF109203X inhibited MEK-Erk activity by 49% at 10 µM but was minimally effective at 1 µM, and Gö 6983 reduced MEK-Erk activity by 90% at 1 µM, these data implicate the contribution of atypical PKCs. The observation that Gö 6983 markedly inhibited MEK-Erk activity at 1 µM contrasted with its effect on mitogenesis, lending credence to the suggestion that long term effects of PKC inhibitors such as that on proliferation, must also take into account other factors described above, in addition to IC_50_ measured using purified proteins. We then determined the effect of PKC inhibition on A-Raf and c-Raf-1 kinase activity using recombinant kinase-dead MEK as substrate. Neither GF109203X nor Gö 6983 significantly influenced A-Raf activity whereas their effect was more pronounced on c-Raf-1 (**Fig. 2C**). However, we have previously shown that while CSF-1 stimulated c-Raf-1 activity, inhibiting c-Raf-1 had no effect on Erk activation [16], consistent with reports that c-Raf-1 could have other functions that are independent of MEK-Erk [42]. Lastly, PKC inhibitors had no effect on Ras activation (**Fig. 2D**). These data indicate that PKC is activating an A-Raf independent pathway downstream of Ras to promote CSF-1 induced MEK-Erk activation.

### CSF-1-stimulated Erk activity is independent of phorbol ester-sensitive PKCs

**Figure 3:**
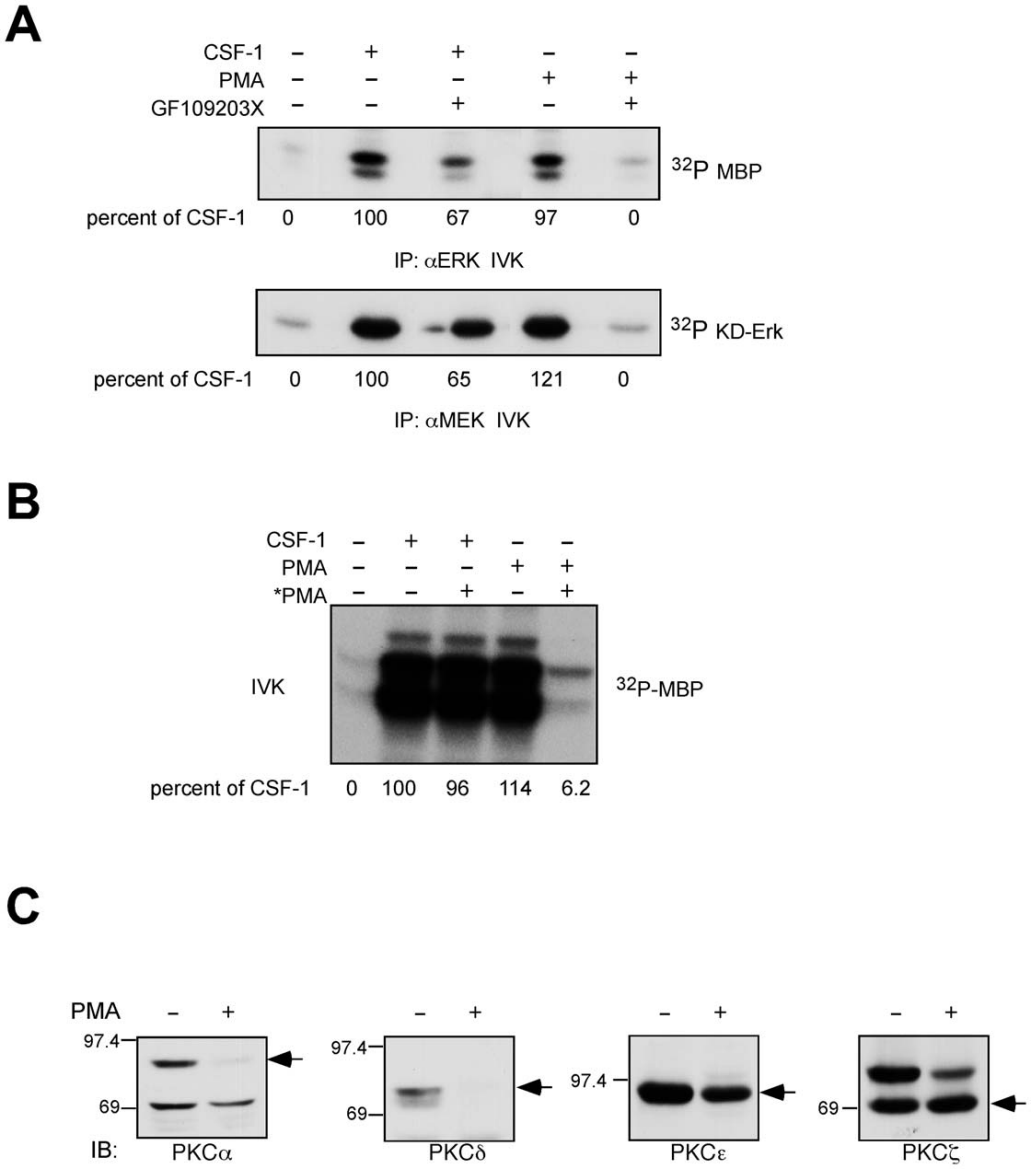
CSF-1-stimulated ERK activity is independent of phorbol ester-sensitive PKCs. (**A**) 32D.R cells were pretreated with 10 µM GF109203X followed by 5 nM CSF-1 or 0.5 µM PMA stimulation for 4 min. Erk and MEK activity were determined in an *in vitro* kinase assay with myelin basic protein (MBP) and KD-Erk as substrate respectively. (**B**) Exponentially growing 32D.R cells were treated with 100 nM PMA for 24 hr to downregulate phorbol ester sensitive-PKCs and continued during the starvation period (designated as *PMA). Cells were either left untreated or treated with 5 nM CSF-1 or 0.5 µM PMA for 4 min. An MBP kinase assay on Erk immunoprecipitates was performed. In (A) and (B), change in phosphorylation is calculated as described in Figure 2. (**C**) Phorbol ester-sensitive PKCs in 32D.R. Total cell lysates were Western blotted with the indicated anti-PKC antibodies in the absence (−) or presence (+) of the immunizing peptide.

The inhibitor studies support a role for PKCs in mediating CSF-1 dependent MEK-Erk activation but did not prove that it was PKCζ. We next set out to exclude an involvement of phorbol ester-sensitive PKCs. Here we used an *in vitro* kinase assay to directly assay Erk and MEK activity (**Fig. 3A**). Both CSF-1 and PMA strongly induced MEK and Erk activity towards an exogenous substrate. Whereas GF109203X was completely inhibitory towards PMA stimulated MEK and Erk activity, in agreement with results in Fig. 2A, it only partially reduced CSF-1 induced MEK and Erk activity. Cells were then treated with PMA for 24 hours to downregulate PMA-sensitive PKCs, starved and acutely stimulated with either CSF-1 or PMA. Downregulating PMA-sensitive PKCs had no effect on CSF-1-mediated ERK activity whereas PMA-induced ERK activity was almost completely abolished (**Fig. 3B**). We next determined which PKC isoforms were susceptible to PMA downregulation (**Fig. 3C**). When cells were exposed to long-term PMA treatment, PKCα and PKCδ disappeared whereas PKCε was downregulated by 34% and there was no effect on PKCζ. Thus, CSF-1-induced ERK activity did not involve PKCα or PKCδ but PKCε and PKCζ remain potential regulators of the ERK pathway.

### CSF-1 stimulates PKCζ kinase activity but has no effect on PKCε translocation

**Figure 4:**
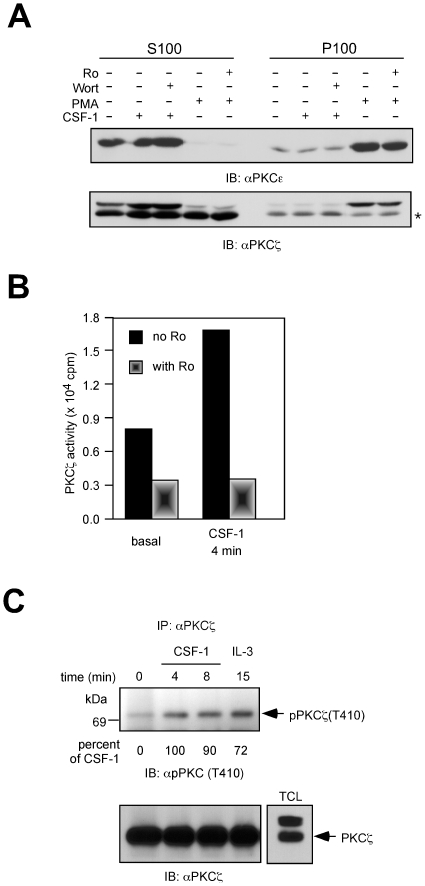
CSF-1 increases PKCζ activity but does not induce PKCε membrane translocation. (**A**) Subcellular fractionation. 32D.R cells were starved, pretreated or not with wortmannin (200 nM) or Ro-31-8220 (30 µM) before stimulation with 5 nM CSF-1 or 0.5 µM PMA for 4 min. Homogenates were separated into cytosolic (S100) and particulate (P100) fractions as described in Methods. 75 µg of each fraction was analyzed by Western blotting with anti-PKCε or PKCζ antibodies. Asterisk indicates the PKCζ protein. (**B**) CSF-1 stimulated PKCζ kinase activity in an *in vitro* kinase assay. Ro-31-8220 was included in parallel experiments. (**C**) CSF-1 stimulated PKCζ phosphorylation. Lysates from 32D.R cells treated as indicated were immunoprecipitated with an anti- PKCζ antibody and blotted with a phospho- PKCζ-T410 antibody. Phosphorylation changes are calculated as described in Figure 2. For reference, at 4 min, CSF-1 stimulated a 7-fold increase in T410 phosphorylation.

Growth factor or PMA-mediated signaling to conventional and novel PKCs can be inferred from translocation of the PKC isoform from cytosol to membrane. We determined if CSF-1 signaled to PKCε, using a membrane translocation assay. Western blotting of equal amounts of cytosolic and membrane fractions showed that in unstimulated cells, PKCε was predominantly cytosolic (**Fig. 4A**). Treatment with CSF-1 for 4 min, the same conditions that induced maximal ERK activation, did not result in PKCε translocation to the membrane. In contrast, PMA induced a marked increase in membrane PKCε immunoreactivity. There was no detectable translocation of PKCζ to the membrane in response to either CSF-1 or PMA. Inhibiting PKC activity with Ro-31-8220 did not influence PMA-induced membrane translocation indicating that membrane recruitment is independent of PKC activity. Similarly, inhibiting PI3K activity with wortmannin that would prevent PDK-1 recruitment to the membrane [43] also had no impact. The upper band recognized by the anti- PKCζ antibody has been proposed to be PKCα by others [44]. Our findings support this hypothesis since PMA induced membrane localization. Curiously, this band was not completely downregulated by PMA even though the PKCα immunoreactive protein was completely degraded (Fig. 3C).

We measured activation of PKCζ by an immune complex kinase assay using the PKCε pseudosubstrate peptide as substrate. This is reported to be the best *in vitro* substrate for PKCζ [45], [46]. CSF-1 stimulated a 2-fold increase in PKCζ kinase activity (**Fig. 4B**). A similar, 2-fold increase in activity was obtained using MBP, another commonly used substrate (data not shown). When 1 µM Ro-31-8220 was included in the kinase reaction, the activity was reduced to below basal levels. Atypical PKCs are phosphorylated by PDK-1 on Thr 410 which is in the activation loop [22]. We used an antibody that detects phosphorylated Thr 410 and found that CSF-1 significantly increased PKCζ Thr 410 phosphorylation (**Fig. 4C**). For comparison, we also determined that IL-3 increased PKCζ Thr 410 phosphorylation, since 32D.R cells are IL-3 dependent. Taken together, our findings support the possibility that PKCζ, rather than PKCε contributed towards CSF-1-induced ERK activation.

### Dominant-negative PKCζ inhibits Erk activity stimulated by either CSF-1 or PMA and constitutively active PKCζ activates Erk in the absence of growth factor

**Figure 5:**
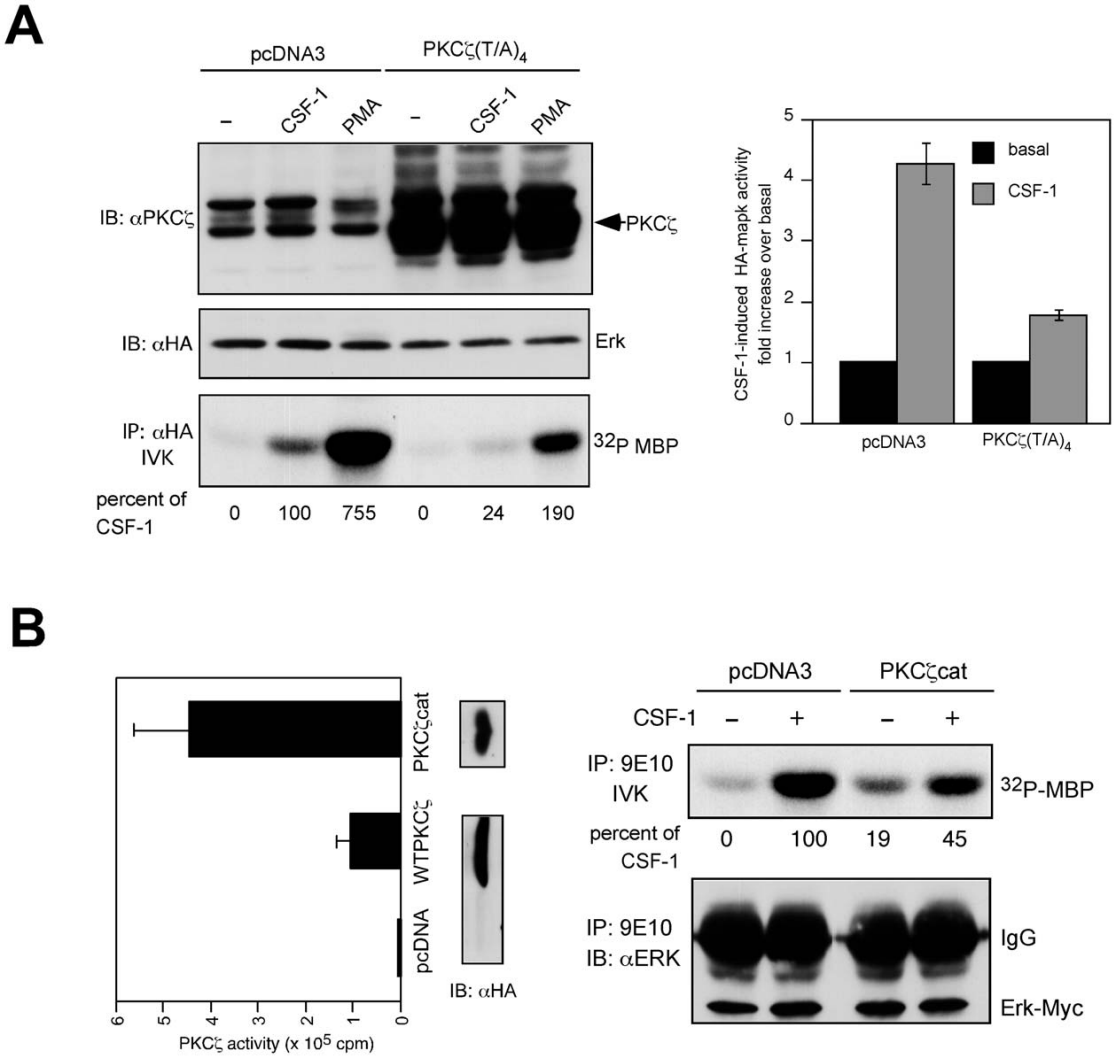
The effect of dominant-negative and constitutively active *PKCζ* on ERK activation. (**A**) Dominant-negative PKCζ. 32D-CSF-1R cells were transfected with 4 µg of HA-ERK and 10 µg of either pcDNA or PKCζ (T/A)_4_. 24 hr later, cells were starved and stimulated with 10 nM CSF-1 or 0.5 µM PMA for 4 min. Expression of transfected proteins was confirmed by blotting with anti- PKCζ (top) or anti-HA (middle) antibodies. HA immunoprecipitates were analyzed for MBP kinase activity (bottom). Phosphorylation changes are calculated as described in Figure 2. In the experiment shown, CSF-1 induced a 3.9-fold increase in MBP phosphorylation over unstimulated cells. Panel on the right shows the averaged results from 3 independent transfections. Data are expressed as the mean ± SD. (**B**) Constitutively active PKCζ. Cells were transfected with 10 µg of Myc-ERK and 40 µg of either pcDNA, HA-WT-PKCζ or HA-PKCζcat. They were treated as described in (A). (left) PKCζ *in vitro* kinase activity. PKCζ was immunoprecipitated using an HA antibody and subjected to an *in vitro* kinase activity with PKCε pseudosubstrate peptide as a substrate. Shown are the means ± SD (n = 3). (right) Lysates containing approximately equivalent amounts of Myc-ERK were analyzed for MBP kinase activity in anti-Myc immunoprecipitates. Basal MBP phosphorylation in cells transfected with PKCζcat was 1.8 fold over that in cells transfected with an empty vector.

We further investigated the involvement of PKCζ by determining the effect of transient expression of a dominant-negative PKCζ [30]. An empty vector or a PKCζ construct in which four threonines in the activation loop (including Thr 410, the PDK1 phosphorylation site) have been replaced with alanines was transiently co-expressed with HA-Erk. CSF-1 and PMA-induced MBP kinase activity in HA immunoprecipitates was assayed 24 hrs later. Western blot of total cell lysates with anti- PKCζ antibody verified overexpression of PKCζ (T/A)_4_ (**Fig. 5A**). The transfected PKCζ co-migrated with the faster-migrating endogenous band, supporting our earlier conclusion regarding its identity. PKCζ (T/A)_4_ suppressed CSF-1-stimulated HA-Erk activity (average inhibition of 76%±4%, n = 3, *p*<0.005) but it also suppressed PMA-induced ERK activity by an equivalent amount (Fig. 5A). The lack of specificity of the dominant-inhibitory actions of PKCζ (T/A)_4_ towards the different PKC isoforms has been observed by others and may be a common property of many dominant-negative PKC mutants, reflecting a common mechanism of activation (Thr 410 phosphorylation by PDK-1) and sequestration of upstream activators [30]. Nevertheless, since CSF-1 dependent MEK-Erk activation did not depend on PMA-sensitive PKCs, the inhibition by dominant-negative PKCζ therefore supports a role for PKCζ.

In another approach to investigate if PKCζ could contribute to ERK activation, we used HA-tagged constitutively active PKCζ containing only the catalytic domain (PKCζcat). First, we determined the kinase activity of transfected HA-tagged wildtype (WT) and PKCζcat. As shown (**Fig. 5B**), only HA immunoprecipitates from cells transfected with PKCζ but not with control vector showed kinase activity. PKCζcat exhibited considerably higher kinase activity when compared to WT- PKCζ. PKCζcat was transiently co-expressed with Myc-tagged Erk (Fig. 5B). MBP kinase assay was performed on Myc immunoprecipitates and showed that basal ERK activity was increased in cells transfected with PKCζcat over that with empty vector control (Fig. 5B, fold increase over vector control in unstimulated cells: experiment #1 = 1.8, experiment #2 = 3.5). Although the increase was not marked, it is consistent with published reports [47] and show that in 32D.R cells, PKCζ can regulate Erk activity. The findings with dominant-negative and constitutively active PKCζ support the PKC inhibitor studies and implicate a role for PKCζ in CSF-1 stimulated Erk activation.

### TNFα but not CSF-1 activates the NF-κB pathway in 32D.R cells

**Figure 6:**
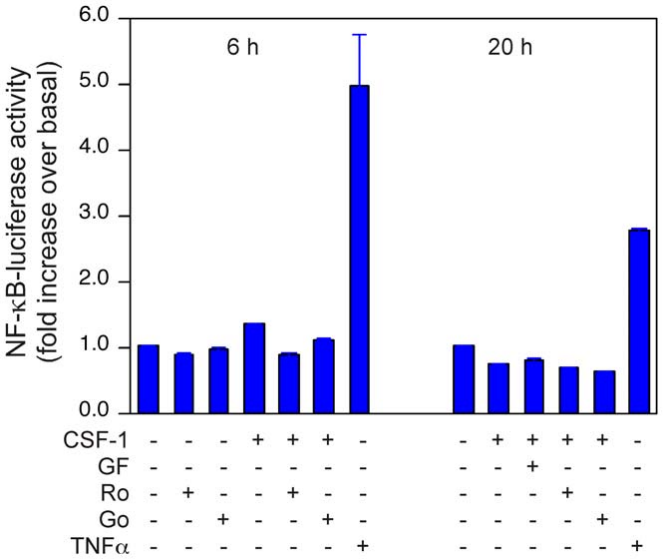
CSF-1 does not activate an NF-κB luciferase reporter in 32D.R cells. A 32D.R line stably expressing an NF-κB luciferase reporter was treated as indicated and lysates subjected to a luciferase assay at 6 h or 20 h after stimulation. CSF-1 was used at 5 nM and TNFα at 20 ng/mL. Inhibitors were used at GF109203X (10 µM), Ro-3108220 (1 µM) and Gö 6893 (5 µM).

Since PKCζ appeared to play a role in CSF-1 mediated mitogenesis and PKCζ has been shown to activate the NF-κB pathway which regulates genes involved in cell proliferation [48], we examined if CSF-1 stimulated NF-κB activity. Transient transfection with an NF-κB reporter did not show any detectable activity (data not shown). To confirm this finding, we established a stable 32D.R cell line expressing an NF-κB reporter. Although TNFα robustly stimulated NF-κB reporter activity, CSF-1 had no effect (**Fig. 6**). These findings demonstrated that PKCζ does not use the NF-κB pathway to promote CSF-1 induced mitogenesis.

### Overexpression of WT- PKCζ in 32D.R cells increased Erk activity and sensitivity to CSF-1 induced proliferation

**Figure 7:**
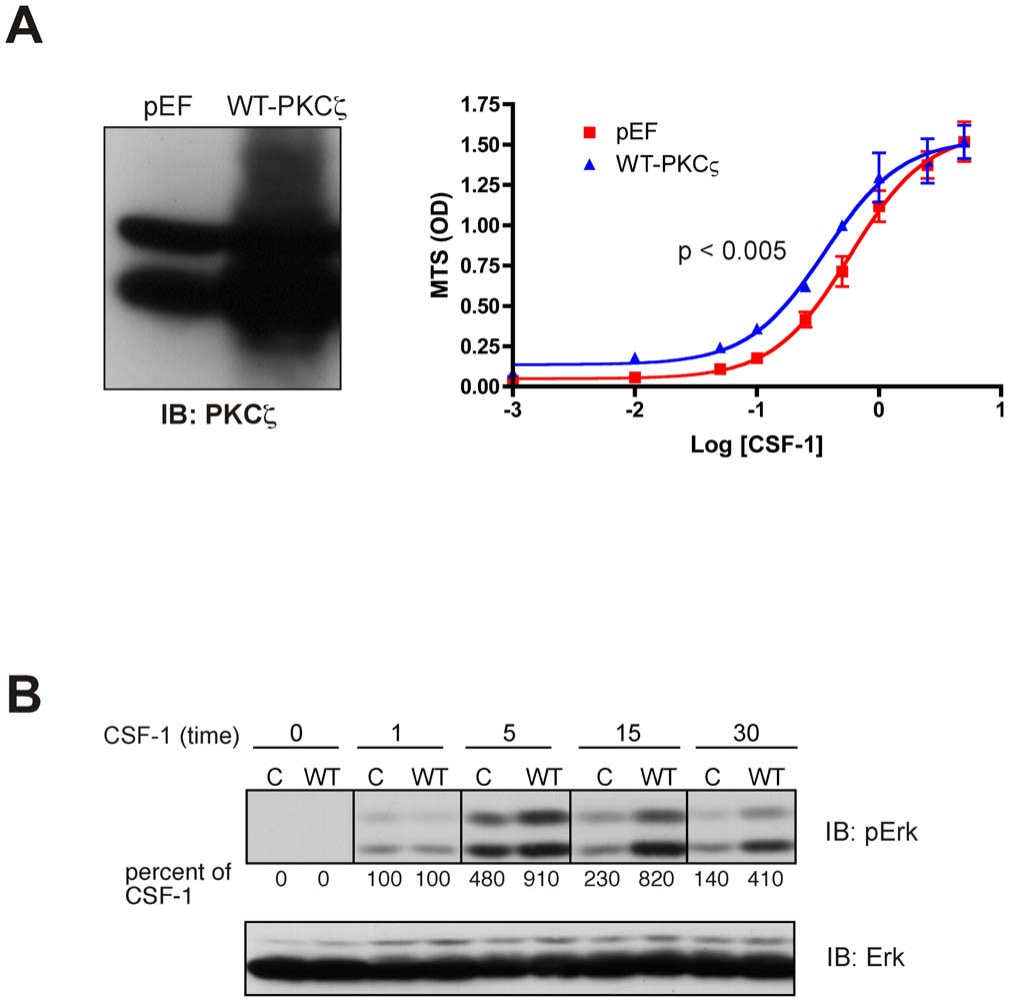
WT-PKCζ overexpression enhances CSF-1 dependent proliferation and Erk phosphorylation. (**A**) (left) PKCζ protein expression in 32D.R cells transfected with empty vector (pEF) or WT- PKCζ. (right) CSF-1 dose responsive MTS assay. The two curves were statistically different. CSF-1 concentrations were in nM. (**B**) Erk phosphorylation in control 32D.R cells (C) or 32D.R cells overexpressing WT- PKCζ (WT) in response to CSF-1 stimulation for the indicated times. Lysates were immunoblotted with anti-phospho-Erk (top) or total Erk (bottom) antibodies. Phosphorylation changes are calculated as described in Figure 2 relative to that determined for control cells stimulated by CSF-1 for 1 min (fold increase is 4.6).

We established a stable cell line overexpressing WT- PKCζ (**Fig. 7A**). We were unsuccessful in establishing stable lines expressing PKCζcat, suggesting that unregulated PKCζ activity may be detrimental to 32D.R cell survival. CSF-1 dose response MTS results showed a shift in EC_50_ from 0.58 nM in vector-transfected cells to 0.36 nM in WT- PKCζ overexpressing cells (*p* = 0.0005) indicating enhanced sensitivity to CSF-1 provoked mitogenesis. Importantly, CSF-1 induced Erk phosphorylation was also increased in cells overexpressing WT- PKCζ and this enhancement was observed at 5 min and beyond (**Fig. 7B**). Altogether, our results in 32D.R cells support a role for PKCζ mediating a part of the CSF-1 dependent Erk activity and mitogenic response.

### PKC inhibition paradoxically enhanced CSF-1 induced MEK-Erk activity in BMMs

**Figure 8:**
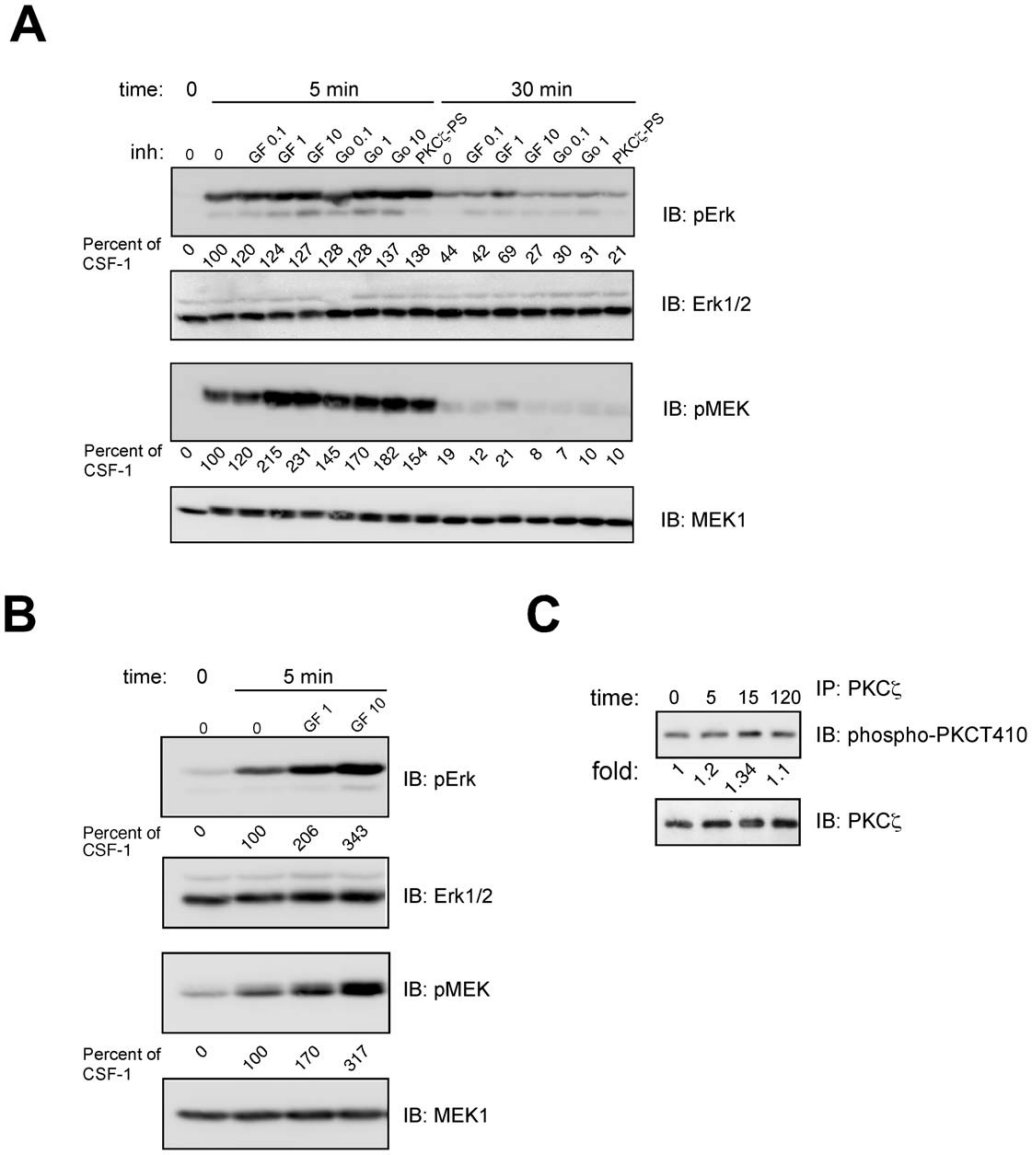
The effect of PKC inhibitors on MEK-Erk activation in bone marrow derived macrophages. (**A**) BMMs were starved in the absence of CSF-1 for 12 h, inhibitors were added or not for 1 h followed by stimulation with 5 nM CSF-1. Inhibitor concentrations used were: GF109203X (GF) at 0.1, 1 and 10 µM; Go 6983 (Go) at 0.1, 1 µM and PKCζ-PS peptide at 10 µM. Lysates were immunoblotted with phospho-Erk and total Erk antibodies (top 2 panels) or with phospho-MEK and total MEK antibodies (bottom 2 panels). Phosphorylation changes are shown as a percent of that determined for CSF-1 stimulation at 5 min in the absence of inhibitors. For the data shown, CSF-1 stimulated MEK and Erk by 46- and 62-fold over untreated cells. (**B**) BMMs were starved in serum free media for 6 h, 1 or 10 µM GF109203X was added as shown followed by CSF-1 stimulation for 5 min. (**C**) PKCζ phosphorylation. (Top) PKCζ was immunoprecipitated with a rabbit polyclonal antibody and blotted with an antibody that recognizes PKCζ phospho-Thr 410. (Bottom) The blot was stripped and reprobed with a monoclonal antibody that recognizes PKCζ. Shown are representative results from one of two experiments performed. Immunoblot images in Figure 8 were captured digitally using the AlphaImager Gel Imaging System (FluorChem Q, Cell Biosciences, Santa Clara, CA).

Under the influence of CSF-1, myeloid progenitors differentiate to macrophages [1]. We were interested in the role of PKCζ in CSF-1 dependent Erk activation and proliferation in macrophages. However, the 32D.R cell line does not differentiate to mature macrophages in response to CSF-1 as demonstrated by flow cytometric analysis (Fig. S1), and the absence of adherence to plastic. We therefore utilized bone marrow derived macrophages (BMMs). Although it would have been preferable to use a single system to assess the role of PKC in progenitors and mature macrophages, obtaining primary myeloid progenitors in sufficient numbers for biochemical assays of the type described is impractical. BMMs were starved in the absence of CSF-1 for 12 hr and then re-stimulated with CSF-1. At concentrations of GF109203X and Gö 6850 that had significantly inhibited CSF-1 induced MEK-Erk activity in 32D.R myeloid progenitors, we saw a paradoxical increase in CSF-1 dependent MEK and Erk phosphorylation in BMMs (**Fig. 8A**). Pretreatment with the PKCζ pseudosubstrate peptide resulted in a similar increase in MEK and Erk phosphorylation. To determine if starvation conditions could be a factor, we starved BMMs in serum-free media for 6 hrs before re-stimulation with CSF-1 in the presence or absence of GF109203X. A similar increase in MEK-Erk phosphorylation was observed (**Fig. 8B**). In BMMs, PKCζ Thr 410 phosphorylation was minimally increased by CSF-1 treatment (Fig. 8C). These data indicate that PKCs impact the MEK-Erk pathway differentially in mature macrophages compared to progenitors. Since the increase in MEK-Erk phosphorylation was also seen at inhibitor concentrations that target conventional and novel PKCs, it would appear that PKC inhibitors are directed at a common mechanism that normally negatively regulates the MEK-Erk pathway.

### The effect of PKC inhibition on CSF-1 supported BMM proliferation

**Figure 9:**
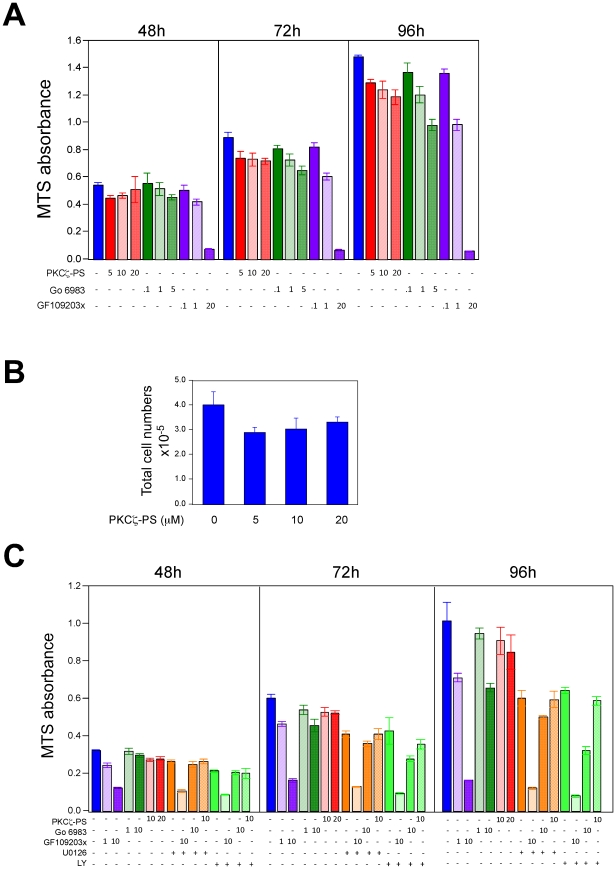
The effect of PKC inhibitors on bone marrow derived macrophage proliferation. (**A**) BMMs were treated with the inhibitors indicated in the presence of 5 nM CSF-1 and MTS activity assayed at 48 h, 72 h and 96 h. Similar results were obtained with 0.25 nM and 1 nM CSF-1 (not shown). (**B**) BMMs were treated with PKCζ PS peptide inhibitor in the presence of CSF-1 and cell counts determined at 96 h. (**C**) The experiment was performed as described in (A). The concentrations shown are in µM. U0126, 10 µM; LY, 5 µM LY294002.

In the absence of CSF-1, BMMs undergo cell death within 24 h hence all of our proliferation assays were performed in the presence of CSF-1. Treatment of BMMs with the PKCζ pseudosubstrate peptide reduced proliferation by a small amount (15–20%) as assessed by MTS activity and cell counts (**Fig. 9A and B**). To determine if PKCζ synergized with other pathways to promote proliferation in BMMs, we used U0126 to inhibit MEK-Erk and LY294002 to inhibit PI3K. After 4 days, U0126 or LY294002 treatment reduced CSF-1 supported mitogenesis by 40%; including the PKCζ pseudosubstrate did not lead to additional reduction (**Fig. 9C**). These data indicate that PKCζ does not have a major role in supporting proliferation in BMMs. We also investigated the impact of GF109203X and Gö 6983 inhibition. In contrast to 32D.R cells, GF109203X was more potent than Gö 6938 in reducing mitogenesis (Fig. 9A and 9C). At 1 µM, GF109203X reduced mitogenesis by 40% compared to <10% for 1 µM Gö 6850, underscoring the contribution of cellular factors in addition to IC_50_ to long-term effects of PKC inhibitors. Interestingly, ≥10 µM GF109203X dramatically reduced proliferation, not seen in 32D.R cells, suggesting that GF109203X may target an as yet unknown protein in BMMs. At 10 µM, Gö 6983 synergized with either U0126 or LY294002 to inhibit proliferation, with the combination of LY294002 and Gö 6983 being more potent. These data suggest that in contrast to 32D.R myeloid progenitors, PKCζ has less of a role in promoting proliferation in BMMs but conventional/novel PKCs cooperate with MEK-Erk and PI3K to support BMM proliferation.

## Discussion

PKCs play pleiotropic roles in many cellular processes. How PKCs contribute to CSF-1 dependent functions is not well characterized. In this study, we addressed two questions using the 32D.R myeloid progenitor cell line and primary mouse BMMs : 1) Is PKC an activator of the MEK-Erk pathway; 2) Does PKC contribute towards CSF-1 dependent proliferation? In 32D.R cells, we presented evidence in support of a role for the atypical PKC, PKCζ, in CSF-1 dependent activation of MEK-Erk and mitogenesis. (1) Downregulation of PKCα and PKCδ by chronic phorbol ester treatment had no effect on CSF-1 mediated MEK-Erk activation, excluding a role for these enzymes. (2) The novel PKC, PKCε, which was expressed in these cells, was partially downregulated by chronic phorbol ester treatment but was not stimulated by CSF-1 in a membrane translocation assay and was unlikely to be involved. (3) PKCζ kinase activity and Thr 410 phosphorylation were increased by CSF-1. (4) At concentrations of GF109203X and Gö 6983 that should inhibit the kinase activity of PKCζ, CSF-1 induced MEK-Erk activity was significantly reduced. This conclusion was supported by the results of transient overexpression of dominant-negative or constitutively active PKCζ on Erk activity. Moreover, stable overexpression of WT- PKCζ increased the magnitude and duration of Erk activation. (5) At concentrations of PKC inhibitors that reduced CSF-1-supported Erk activation, mitogenesis was also suppressed. This was corroborated using a PKCζ specific inhibitory peptide and by stable overexpression of WT- PKCζ. In contrast, in BMMs, PKC inhibition increased CSF-1 dependent MEK-Erk activation indicating a different role for PKCs compared to immature cells. PKCζ was not a major contributor to proliferation but conventional/novel PKCs appeared to collaborate with the Erk and PI3K pathways to support mitogenesis.

Previously, we showed in 32D.R cells that CSF-1 activated MEK-Erk using both an A-Raf-dependent and an A-Raf independent pathway that did not involve c-Raf-1 [16]. Since PKC inhibitors had no effect on A-Raf activity, we believe that PKCζ activated MEK-Erk independent of A-Raf. CSF-1 increased Thr 410 phosphorylation on PKCζ which is PDK1 mediated [26], [49]. PDK1 is constitutively activated but depends on PI3K for membrane localization [43]. Although we did not detect CSF-1-induced translocation of PKCζ to the membrane, about 12% of total PKCζ was found in the particulate fraction and this population is poised to interact with PDK1. PDK1 is also distributed throughout the cell [43] so that PDK-1 mediated phosphorylation of the active site in PKCζ can also occur in the cytosol. Regardless of where PKCζ is phosphorylated in 32D.R cells, CSF-1 induced a significant increase in Thr 410 phosphorylation. How PKCζ activates MEK-Erk is not well understood. The epidermal growth factor induces the association of PKCζ with MEK5, a pathway that is important for mitogenic signaling [50]. However the docking site identified in MEK5 for PKCζ is absent in MEK1/2, raising the question of whether there is another MEK kinase that is stimulated by PKCζ.

In BMMs, inhibitors that target conventional/novel/atypical PKCs all appeared to upregulate CSF-1 supported MEK-Erk activity. Our observations are consistent with PKC inhibition relieving negative regulation of MEK-Erk signaling. MAPKs and MEK1/2 are dephosphorylated by dual specific phosphatases (DUSPs) and protein phosphatases 2A respectively [51]. PKCs could promote the activation of these phosphatases. Our findings extend those reported in an earlier study on BMMs where it was found that CSF-1 induction of DUSP1 (MKP-1) is dependent on PKCε [52]. These authors reported that GF109203X pretreatment prolonged Erk phosphorylation but did not enhance its degree of activation, which is consistent with the timeframe of DUSP1 induction. In contrast, we saw an increase in MEK-Erk activation at 5 min that is already dissipated at 30 min. Since DUSP1 is induced closer to 30 min than 5 min, DUSP1 is unlikely to be responsible for the increase in Erk phosphorylation in the presence of PKC inhibitors; also DUSP1 is not known to dephosphorylate MEK. A second possibility is that PKC activity is necessary for the action of a negative regulator upstream of MEK. The negative regulator could be directed at the MEK kinase or a scaffold such as KSR that brings the members of the MEK kinase-MEK-Erk module into close proximity [53]. Our findings contrast with those in the BAC1.2F5 macrophage cell line where no effect of GF109203X on CSF-1 stimulated MEK and Erk kinase activity was observed [54]. Possibly this could reflect a difference in cell type (primary versus cell line) or assay (phosphorylation versus kinase activity), although we have found that dual phosphorylation at the TEY motif as detected by the antibody used in this study to track kinase activity faithfully. More extensive investigations will be required to determine the mechanism of PKC input into Erk activation in BMMs.

Our data show that PKCζ contributes towards CSF-1 mediated mitogenesis in 32D.R cells, in part through the MEK-Erk pathway. Since PKCζ is known to activate the NF-κB pathway [25], it was surprising to find that CSF-1 did not transactivate an NF-κB reporter construct stably expressed in 32D.R cells whereas TNFα was a potent activator in these cells. PKCζ is thought to phosphorylate the RelA subunit of NF-κB at Ser311 and the phosphorylation assists in setting up CBP recruitment [55]. However other activation steps are necessary and presumably, were not stimulated by CSF-1 in 32D.R cells. In BMMs, PKCζ does not appear to play a major role to support CSF-1 mediated proliferation. Conventional/novel PKCs are more important and PKC inhibitors synergize with MEK and PI3K inhibition in reducing proliferation, indicating PKC, MEK and PI3K to be working on parallel pathways. The impact of PKC inhibitors on mitogenesis in BMMs may have been offset by their simultaneous promotion of MEK-Erk activation, so that the actual contribution of PKC to mitogenesis in BMMs may have been more significant than the data showed. In addition to proliferation, different groups have investigated PKC action in other aspects of mononuclear phagocyte function. PKCζ is said to be important in CSF-1 induced chemotaxis in macrophages [29], RANKL/CSF-1 mediated bone remodeling [56] and PKCδ is important for LPS-induced soluble Flt-1 expression [57]. Additionally PKCs could be involved in myeloid differentiation, primarily involving PKCδ [27], [58].

In summary, we have identified PKCζ as a crucial activator of MEK-Erk in the CSF-1 signaling pathway in myeloid progenitors. In mature macrophages, PKCζ and other PKC members appear to target a common negative regulator of CSF-1-dependent MEK-Erk activity. These findings indicate that the cellular context of CSF-1 signaling must be considered and the role of different PKC subclasses in macrophages warrants an in-depth investigation.

## Supporting Information

Figure S1 **32D.R cells have an immature immunophenotype that is different from bone marrow derived macrophages (BMM).** 32D.R cells were cultured in the presence of CSF-1 or IL-3 before analysis. Day 7 BMMs (see Methods) were used. (**A**) Forward and side scatter profile. (**B**) Staining of 32D.R with isotype control antibodies (isotype), or with CD31 and CD11b. (**C**) Staining of 32D.R with isotype control antibodies (isotype), or with CD31 and Ly6C. (**D**) Staining of BMMs with CD31, CD11b or with CD31, Ly6C.
(TIF)

Methods S1 Supplementary methods.
(DOC)
