# Supplementary figures and images for: The Role of Atypical Protein Kinase C in CSF-1-Dependent Erk Activation and Proliferation in Myeloid Progenitors and Macrophages
Source: PLoS One. 2011 Oct 18;6(10):e25580. doi: 10.1371/journal.pone.0025580 (PMC3196503; doi:10.1371/journal.pone.0025580)

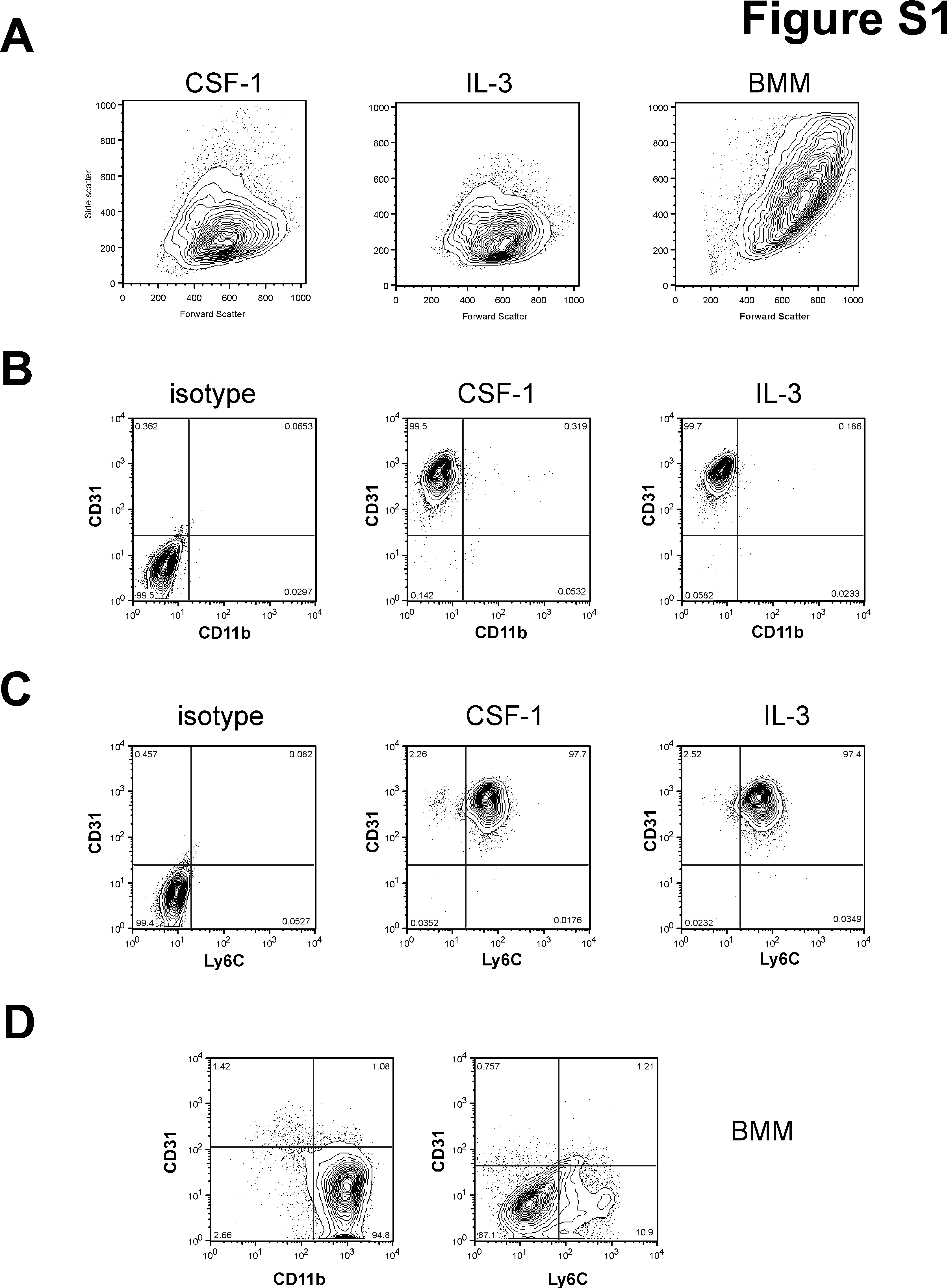

Supplement: Figure S1 — 32D.R cells have an immature immunophenotype that is different from bone marrow derived macrophages (BMM). 32D.R cells were cultured in the presence of CSF-1 or IL-3 before analysis. Day 7 BMMs (see Methods) were used. (A) Forward and side scatter profile. (B) Staining of 32D.R with isotype control antibodies (isotype), or with CD31 and CD11b. (C) Staining of 32D.R with isotype control antibodies (isotype), or with CD31 and Ly6C. (D) Staining of BMMs with CD31, CD11b or with CD31, Ly6C. (TIF) [file pone.0025580.s001.tif]
