# Supplementary material for: The Role of Atypical Protein Kinase C in CSF-1-Dependent Erk Activation and Proliferation in Myeloid Progenitors and Macrophages
Source: PLoS One. 2011 Oct 18;6(10):e25580. doi: 10.1371/journal.pone.0025580 (PMC3196503; doi:10.1371/journal.pone.0025580)
Supplement: Methods S1 — Supplementary methods. (DOC) [file pone.0025580.s002.doc]

**SUPPLEMENTAL METHODS**

**Flow cytometry.** 32D.R cells or bone marrow derived macrophages were washed and then blocked with CD16/CD32 (FcγR II and FcγR III) antibodies (clone 93 from eBioscience, San Diego, CA) for 10 minutes prior to staining with fluorophore-conjugated antibodies for 20 min on ice. Ethidium monoazide (Invitrogen, Carlsbad, CA) was used at the same time to exclude dead cells . Cells were then washed in phosphate buffered saline with 2% fetal bovine serum, fixed in BD Stabilizing Fixative (BD Biosciences, San Jose, CA) and ran on a FACSCalibur (BD Biosciences). 20,000 events were recorded. Antibodies used for flow analysis were purchased from either BD Biosciences or eBioscience: CD11b (clone M1/70), CD31 (MEC13.3) and Ly6C (AL-21). CD11b (or Mac-1) is expressed on more mature myeloid cells and the differential expression of CD31 and Ly6C has been used to differentiate different stages of macrophage development .

1. Perfetto SP, Chattopadhyay PK, Roederer M (2004) Seventeen-colour flow cytometry: unravelling the immune system. Nat Rev Immunol 4: 648-655.

2. de Bruijn MF, Slieker WA, van der Loo JC, Voerman JS, van Ewijk W, et al. (1994) Distinct mouse bone marrow macrophage precursors identified by differential expression of ER-MP12 and ER-MP20 antigens. Eur J Immunol 24: 2279-2284.
